# Defining Disease Modification in IgA Nephropathy: Toward a Paradigm Shift in Management

**DOI:** 10.1016/j.ekir.2025.09.025

**Published:** 2025-09-30

**Authors:** Jonathan Barratt, Laura H. Mariani, Jai Radhakrishnan, Dana V. Rizk, James A. Tumlin, Richard A. Lafayette

**Affiliations:** 1 Department of Cardiovascular Sciences, University of Leicester, Leicester, UK; 2 Division of Nephrology, Department of Internal Medicine, University of Michigan, Ann Arbor, Michigan, USA; 3 Department of Medicine, Vagelos College of Physicians and Surgeons, Columbia University, New York, New York, USA; 4 Division of Nephrology, Department of Medicine, Heersink School of Medicine, University of Alabama at Birmingham, Birmingham, Alabama, USA; 5 Department of Medicine, Division of Renal Medicine, Emory University, Atlanta, Georgia, USA; 6 NephroNet Clinical Trials Consortium, Atlanta, Georgia, USA; 7 Division of Nephrology, Department of Medicine, Stanford University, Stanford, California, USA

**Keywords:** disease modification, glomerular filtration rate, IgA nephropathy, kidney failure, proteinuria, surrogate end point

## Abstract

IgA nephropathy (IgAN) is a rare, chronic, immune-mediated kidney disease characterized by a slow, progressive decline in kidney function. As a disease without an existing cure and a leading cause of chronic kidney disease (CKD) and kidney failure, IgAN requires effective interventions for disease modification. However, identifying interventions as “disease modifying” is challenging in IgAN because of a lack of consensus on the term and uncertainty about suitable markers by which “disease modification” should be defined. This review discusses how “disease modification” could be defined in IgAN, based on the simple premise of the need to preserve nephrons and avoid progression to kidney failure within the patient’s lifetime. In addition, how disease modification can be meaningfully assessed (e.g., the impact of an intervention on mortality and kidney failure, estimated glomerular filtration rate [eGFR], urinary protein or albumin, nonvisible [microscopic] hematuria, and markers of underlying IgAN pathology) is examined. Further, the concept of a multifaceted approach to IgAN management is discussed, targeting both the IgAN-specific processes leading to nephron loss and the generic maladaptive responses to IgAN-induced nephron loss.

IgAN is a rare, chronic, immune-mediated kidney disease that is most common in patients of Asian ancestry, followed by those of European descent.^1^, ^2^, ^3^, ^4^ It is characterized by a slowly progressive decline in kidney function consequent to the mesangial deposition of immune complexes containing galactose-deficient IgA type 1 (Gd-IgA1).^1^, ^2^, ^3^ IgAN places a severe burden on patients. A study of patients with IgAN in the UK National Registry of Rare Kidney Diseases showed that most patients progressed to kidney failure within 20 years of diagnosis.^5^ The same study showed that declines in eGFR ≥ 3 ml/min per 1.73 m^2^/yr in patients in the UK National Registry of Rare Kidney Diseases aged < 40 years were associated with a 100% chance of developing kidney failure within their lifetime (Figure 1).^5^

**Figure 1:**
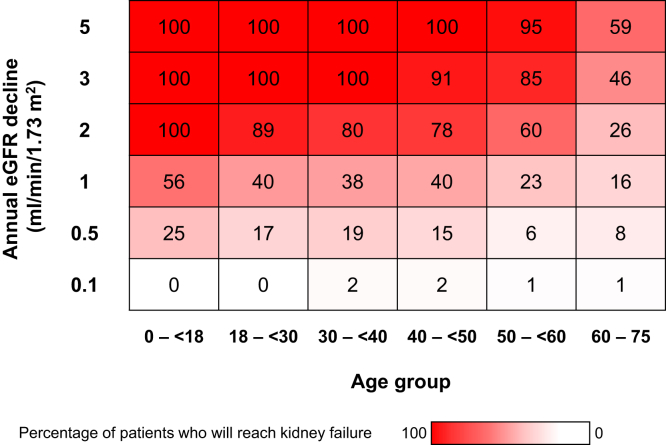
Data from the UK National Registry of Rare Kidney Diseases showing the percentage of patients who will reach kidney failure during their life expectancy on the basis of their eGFR at diagnosis. Life expectancy was based on year of birth and sex. Figure reproduced with permission from Wolters Kluwer Health.^5^ eGFR, estimated glomerular filtration rate.

Understanding the natural history of IgAN highlights the need for disease-modifying therapies. Many patients experience a prolonged subclinical phase marked by hematuria and low-grade proteinuria, with preserved kidney function. Despite this, immune-mediated injury progresses silently, and at least approximately 40% of patients develop kidney failure within 20 years.^5^, ^6^, ^7^, ^8^, ^9^ Even those with initially stable disease may experience gradual nephron loss. This slow but relentless course of disease progression offers a window for intervention aimed at preserving kidney function.^5^ Defining disease modification in IgAN is therefore rooted in the goal of interrupting the decline in function before kidney failure occurs.

The poor prognosis associated with IgAN is in part because of an often delayed diagnosis, with a large proportion of patients presenting with already impaired kidney function.^5^^,^^10^^,^^11^ In the USA, for example, most patients with IgAN present with CKD stage 2 or worse at diagnosis, with a large proportion presenting with an eGFR < 60 ml/min per 1.73 m^2^.^11^^,^^12^ It is generally accepted that an eGFR < 60 ml/min per 1.73 m^2^ equates to a loss of ≥ 50% of nephrons. Given that every nephron lost cannot be regained, the therapeutic window to prevent nephron loss and delay kidney failure is narrow for most patients with IgAN.^13^ Therefore, early intervention in IgAN is needed; however, it remains extremely challenging without effective screening programs and the availability of validated, noninvasive, and IgAN-specific biomarkers.

Terms such as complete and partial remission, induction, and maintenance therapy are commonly used in glomerular diseases.^14^ However, these concepts have not been systematically applied to IgAN. A longstanding lack of safe and effective treatments has meant that induction and maintenance therapy regimens have not evolved in IgAN, although this is likely to change with the approval of new therapies for IgAN. Defining complete and partial remission of IgAN has proved challenging with no internationally accepted definitions used either in clinical trials or clinical practice. This is in part because of lack of validated disease-specific biomarkers to help define remission and the uncertain relationship between proteinuria and hematuria and disease activity, which will be discussed later.

To prevent kidney failure in patients with IgAN, as with all forms of kidney disease, we must address the drivers of continued nephron loss and understand what available treatments can deliver in this regard; that is, their ability to modify the disease. The drivers for nephron loss in IgAN can be separated into 2 major classes as follows:

1. The IgAN-specific, immune-mediated drivers of nephron loss: these comprise the different components of the IgAN-specific pathogenic cascade, commonly described as the “four-hit hypothesis” of IgAN (Figure 2).^15^ An excess of Gd-IgA1 accumulates in the circulation (“Hit 1”), which triggers the production of anti–Gd-IgA1 IgG and/or IgA autoantibodies (“Hit 2”).^3^^,^^16^ These form circulating immune complexes (“Hit 3”), which become deposited in the glomerular mesangium (“Hit 4”).^3^^,^^16^ This deposition leads to mesangial cell activation and the release of proinflammatory and profibrotic mediators, causing glomerular inflammation and scarring, and the progressive loss of kidney function.^16^^,^^17^

**Figure 2:**
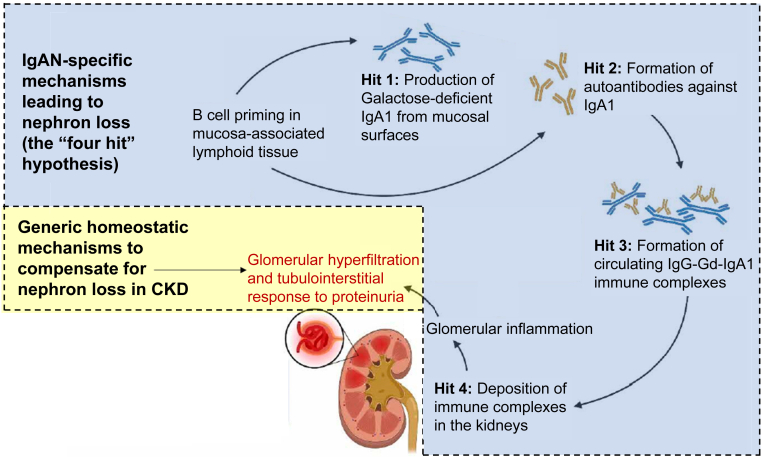
Drivers and mechanisms of nephron loss in IgAN. Figure adapted from Lim *et al.*^15^ CKD, chronic kidney disease; Gd-IgA1, galactose-deficient IgA1; IgA1, Ig A type 1; IgAN, IgA nephropathy.
2. The generic maladaptive responses to IgAN-induced nephron loss: these are common to all forms of CKD and are the result of maladaptive compensatory mechanisms, such as systemic and glomerular hypertension and hyperfiltration, and of the consequences of proteinuria leading to tubulointerstitial injury (Figure 2).^18^, ^19^, ^20^

In this review, we explore how “disease modification” could be defined in IgAN, looking at such definitions in other therapy areas and considering current treatment goals and surrogate end points used in CKD and IgAN that are important to clinicians, regulators, and patients alike.

### Defining and Achieving “Disease Modification” in IgAN

The concept of “disease modification” is firmly established in several therapeutic areas, most notably in rheumatology where the term “disease-modifying antirheumatic drug,” or “DMARD,” has been in use for many decades.^21^, ^22^, ^23^ Although definitions of “disease modification” will vary across therapeutic areas, there are elements of these definitions that are common to all. A conceptual framework for disease modification in systemic lupus erythematosus was developed by van Vollenhoven and colleagues based on the review of definitions across multiple therapy areas, including rheumatologic, neurodegenerative, and respiratory diseases.^22^ The common theme across all fields was that for a treatment to be considered disease modifying, it must have a meaningful effect on the “progressive component or the natural course of the disease” (i.e., slowing or halting disease progression).^22^ This definition is often accompanied by an improvement in the signs and symptoms of the disease.^22^ The authors also included a proposal specific to lupus nephritis; namely, “minimizing disease activity with minimal treatment-associated toxicity and slowing or preventing organ damage progression” (i.e., progression to kidney failure).^22^ In the oncology setting, authors seeking to address disease modification in myelofibrosis highlight that it is “critical for any definition of disease modification to represent true modifiers and mechanisms of improvement, rather than resultant downstream effects.”^24^

In IgAN, the “progressive component or the natural course of the disease” equates to the relentless loss of nephrons over time.^13^ Therefore, in simple terms, “disease modification” in IgAN can be defined as intervening to preserve nephrons or, at the least, slow the rate of their loss, with the intention of avoiding progression to kidney failure within the patient’s lifetime.^13^

Based on the aforementioned 2 main drivers of nephron loss, disease modification in IgAN can be achieved through 2 mostly distinct yet complementary processes as follows: (i) modification of the IgAN-specific pathophysiology and (ii) modification of the generic maladaptive responses to IgAN-induced nephron loss. Modifying both these drivers is essential if kidney failure is to be avoided in patients with IgAN.

Regarding IgAN-specific drivers of nephron loss, the pathophysiologic pathway described by the “four-hit hypothesis” provides multiple targets for disease-modifying interventions. Descriptions of the individual agents that are approved or are in clinical development for use in patients with IgAN are detailed elsewhere.^15^^,^^16^ In summary, there are agents acting early in the pathway to inhibit Gd-IgA1 formation (targeted-release formulation budesonide, B cell−activating factor or a proliferation-inducing ligand inhibitors, anti-cluster of differentiation 38 plasma cell depleters), and others acting later to inhibit glomerular inflammation resulting from immune complex deposition (e.g. complement inhibitors; Table 1^25^, ^26^, ^27^, ^28^, ^29^, ^30^, ^31^, ^32^, ^33^, ^34^, ^35^, ^36^, ^37^, ^38^, ^39^, ^40^, ^41^, ^42^, ^43^, ^44^, ^45^, ^46^, ^47^, ^48^, ^49^, ^50^, ^51^, ^52^, ^53^, ^54^, ^55^, ^56^, ^57^, ^58^, ^59^, ^60^, ^61^).^15^^,^^62^ Systemic corticosteroids are likely to act at multiple levels in the pathogenic cascade, with, at the doses used in IgAN, a predominant action within the kidney to suppress inflammation.^63^^,^^64^ Patients with IgAN have typically already lost a substantial number of nephrons by the time they are diagnosed.^11^^,^^13^ To be maximally effective at avoiding progression to kidney failure during a patient’s lifetime, treatment ideally needs to commence as soon as possible, while there is the maximal number of nephrons to preserve and before downstream irreversible scarring occurs.

**Table 1:** Overview of approved and investigational IgAN therapies^25^, ^26^, ^27^, ^28^, ^29^, ^30^, ^31^, ^32^, ^33^, ^34^, ^35^, ^36^, ^37^, ^38^, ^39^, ^40^, ^41^, ^42^, ^43^, ^44^, ^45^, ^46^, ^47^, ^48^, ^49^, ^50^, ^51^, ^52^, ^53^, ^54^, ^55^, ^56^, ^57^, ^58^, ^59^, ^60^, ^61^

Drivers of nephron loss	Drug class	Drug	Phase of development	eGFR	UACR UPCR	Hematuria	PROs	IgAN Hit 1^a^	IgAN Hit 2^b^	IgAN Hit 3^c^	IgAN Hit 4^d^
Targeting the IgAN-specific, immune-mediated drivers of nephron loss	Mucosal-directed therapy	Nefecon	• FDA full approval in IgAN • EMA full approval in IgAN	Phase 3 data^25^	Phase 3 UPCR and UACR data^25^	Phase 3 data^25^	No effect^26^	Phase 2 and phase 3 data^27^, ^28^, ^29^, ^30^	Phase 3 data^29^^,^^30^	Phase 2 and phase 3 data^27^^,^^29^^,^^30^	Phase 2 and phase 3 data^27^^,^^31^
	APRIL inhibition	Sibeprenlimab	Phase 3	Phase 2 data^32^	Phase 2 UPCR data^32^	Phase 2 data^33^	No data	Phase 2 data^32^	No data	Phase 2 data^34^	No data
		Zigakibart	Phase 3	Phase 1/2 data^35^	Phase 1/2 UPCR data^35^	No data	No data	Phase 1/2 data^35^	No data	No data	No data
	BAFF and APRIL inhibition	Atacicept	Phase 3	Phase 2 data^36^^,^^37^	Phase 2 UPCR data^36^^,^^37^	Phase 2 OLE data^37^	No data	Phase 2 data^36^^,^^37^	Phase 2 data^38^	Phase 2 data^39^	No data
		Telitacicept	Phase 3	Phase 2 data^40^	Phase 2 proteinuria data^40^	No data	No data	Phase 2 data^40^	No data	Phase 2 data^40^	Phase 2 data^40^
		Povetacicept	Phase 3^41^	No data	Phase 1/2 single-arm UPCR data^42^	Phase 1/2 data^42^	No data	Phase 1/2 single-arm data^42^	No data	No data	No data
	Systemic glucocorticoids	Methylprednisolone	N/A	Slope data^43^^,^^44^	Short-term proteinuria data^43^^,^^44^	Data available^43^	No data	No data	No data	No data	Data available^45^
	Alternative pathway complement inhibition	Iptacopan	FDA accelerated approval in IgAN	No data	Phase 3 UPCR data^46^	Phase 3 data	No data	No data	No data	No data	Interim phase 3 data^46^
		Sefaxersen (RO7434656/IONIS-FB-LRx)	Phase 3^47^	Phase 2 data^48^^,^^49^	Phase 2 proteinuria data^48^^,^^49^	Phase 2 data^49^	No data	No data	No data	No data	Phase 2 data^49^
		ARO-C3	Phase 1/2^50^,^51^	No data	No data	No data	No data	No data	No data	No data	No data
	Final common pathway complement inhibition	Cemdisiran	Phase 2	Exploratory phase 2 data^52^	Phase 2 UPCR data^52^	Phase 2 data^52^	No data	No data	No data	No data	Phase 2 data^52^
		Ravulizumab	Phase 2	Phase 2 data^53^^,^^54^	Phase 2 proteinuria data^53^^,^^54^	Phase 2 data^54^	No data	No data	No data	No data	Phase 2 data^54^
		Avacopan	Phase 2	No long-term data^55^	Phase 2 single-arm UPCR and UACR data^55^	No data	No data	No data	No data	No data	Phase 2 single-arm data^55^
Targeting the generic responses to IgAN-induced nephron loss	ACEis, ARBs	Multiple	N/A	Reference	Reference	No data	No data	No data	No data	No data	No data
	DEARA	Sparsentan	• FDA full approval in IgAN • EMA full approval in IgAN	Phase 3 slope data,^56^ RWE eGFR^57^	Phase 3 UPCR and UACR data,^56^ RWE UPCR and UACR^57^	No data	No data	No data	No data	No data	Data inconclusive^e^^,^^58^
	ERA	Atrasentan	FDA accelerated approval in IgAN	No data	Interim phase 3 UPCR data^59^	No data	No data	No data	No data	No data	No data
	SGLT2i	Dapagliflozin	• FDA full approval in CKD • EMA full approval in CKD	Phase 3 slope data (patients with IgAN)^60^	Phase 3 UACR data (patients with IgAN)^60^	No data	No data	No data	No data	No data	No data
		Empagliflozin	• FDA full approval in CKD • EMA full approval in CKD	Phase 3 slope data (patients with IgAN)^61^	Phase 3 UACR data (patients with IgAN)^61^	No data	No data	No data	No data	No data	No data

ACEi, angiotensin-converting enzyme inhibitor; APRIL, a proliferation-inducing ligand; ARB, angiotensin receptor blocker; BAFF, B cell−activating factor; CKD, chronic kidney disease; DEARA, dual endothelin-angiotensin receptor antagonist; eGFR, estimated glomerular filtration rate; EMA, European Medicines Agency; ERA, endothelin receptor antagonist; FDA, US Food and Drug Administration; Gd-IgA1, galactose-deficient IgA type 1; IC, immune complex; IgA1, IgA type 1; IgAN, IgA nephropathy; N/A, not applicable; OLE, open-label extension; PRO, patient-reported outcome; RWE, real-world evidence; SGLT2i, sodium-glucose cotransporter 2 inhibitor; UACR, urine albumin-to-creatinine ratio; UPCR, urine protein-to-creatinine ratio.

a Hit 1 was defined as agents that target the production and accumulation of Gd-IgA1 in the systemic circulation, measurable through levels of circulating Gd-IgA1.

b Hit 2 was defined as agents that target the production of IgG and IgA antibodies, including IgG directed to Gd-IgA1. It was measurable through circulating levels of IgA and IgG antibodies.

c Hit 3 was defined as agents that target the formation of immune complexes (IgA1-IgA/IgG-IC) via the binding of circulating IgG/IgA to Gd-IgA1 and was measurable through circulating levels of immune complexes.

d Hit 4 was defined as agents that target the deposition of immune complexes (IgA-IgA1/IgG-IC) in the glomerulus and was measurable through the downstream effects that lead to mesangial activation, proliferation, inflammatory cell recruitment, and cytokine and extracellular matrix component secretion and complement activation.

e Owing to the small patient number and lack of a control group in the SPARTAN trial, more data are needed.

Modifying the generic maladaptive responses to IgAN-induced nephron loss includes the reduction of systemic and glomerular hypertension and hyperfiltration, and the minimization of proteinuria, as is recommended for all forms of CKD. Optimized supportive care with lifestyle modification, blood pressure control, renin-angiotensin system inhibition with maximally tolerated doses of angiotensin-converting enzyme inhibitors or angiotensin receptor blockers have been the most commonly employed approaches to achieve this.^14^ More recent studies have identified the kidney-protective properties of sodium-glucose cotransporter 2 inhibition and endothelin receptor antagonism in patients with CKD, including patients with IgAN (Table 1).^56^^,^^60^^,^^61^ Research with mineralocorticoid receptor antagonism in patients with IgAN is ongoing (NCT05047263).^65^ However, though these approaches are an essential component of any IgAN disease-modification strategy (and a proportion of patients will experience a stabilization in disease progression with these approaches alone in the medium term), they do not address the upstream immune-related events that lead to nephron loss in IgAN. Therefore, these approaches are unlikely to be sufficient to prevent kidney failure during the patient’s lifetime in those with ongoing immune-mediated damage.^66^^,^^67^

Although IgAN-specific immune-mediated injury is a critical target, particularly early in the disease course, the downstream maladaptive responses common to all CKD—such as hypertension, hyperfiltration, and tubulointerstitial fibrosis—also contribute significantly to nephron loss and become increasingly dominant as the disease progresses.^68^ In clinical practice and trials, the therapeutic effects of many interventions likely reflect a composite impact on both pathways, which are difficult to disentangle. Therefore, both components must be considered in disease modification strategies, with the relative importance of each likely varying by disease stage and individual patient characteristics.

### How Can Disease Modification be Measured in IgAN?

How disease modification can be defined and achieved in IgAN leads to the question of how it can be measured. The earliest changes that could theoretically be detected would be in IgAN-specific biomarkers that are associated with IgA deposition in the kidneys and IgAN-specific, immune-mediated nephron loss. Hematuria, albuminuria, and proteinuria appear earlier in the course of the disease than a change in eGFR,^69^^,^^70^ which is subsequently observed as more nephrons are lost. The ultimate conclusion of this pathogenic process is kidney failure (and death without renal replacement therapy).^13^ However, there are a number of practical factors relating to the use of these outcomes that must be discussed.

Existing clinical trials in IgAN commonly use surrogate end points such as percent reduction of protein in the urine and eGFR slope over time.^25^^,^^46^^,^^56^^,^^59^, ^60^, ^61^ However, definitions vary; for example, whether reduction of urinary protein is measured as absolute or percentage change, or whether eGFR slope refers to total versus chronic slope or change from baseline.^25^^,^^46^^,^^56^^,^^59^, ^60^, ^61^ Similarly, assays used to measure IgAN-specific biomarkers (e.g., Gd-IgA1, anti–Gd-IgA1 antibodies, immune complexes) lack standardization and validation across studies.^71^, ^72^, ^73^, ^74^ Without harmonized definitions and assay platforms, comparisons across trials and translation into clinical practice remain difficult. Future consensus efforts are needed to define uniform, clinically meaningful thresholds and validate biomarker assays for regulatory and clinical use. Ideally, future treatment effectiveness will be measured against the recently revised Kidney Disease: Improving Global Outcomes guidelines, which indicate that the treatment goal in patients with IgAN at risk of progressive loss of kidney function is to reduce the rate of loss of kidney function to the physiological state (i.e., < 1 ml/min/yr for most adults).^75^

### Kidney Failure and Mortality

Although limited, there are data suggesting that IgAN is associated with an increased risk of death.^76^ Therefore, as the worst possible outcome of IgAN, a positive impact of treatment on death rates would naturally be the most impactful measure of disease modification from a patient perspective. Progression to kidney failure is the principal driver of mortality in these patients^76^; thus, a combined approach of addressing both outcomes would be an appropriate measure of disease modification for both interventions targeting IgAN-specific and generic CKD drivers of nephron loss. However, “hard” clinical end points such as death and kidney failure are infrequently observed in IgAN clinical trials. Furthermore, there is an obvious need to assess disease modification well before these outcomes occur and to intervene as early as possible to prevent nephron loss.^5^ Therefore, though mortality and kidney failure are clinically meaningful measures of disease modification in IgAN, they are not practical to use as outcome measures to assess the response to an intervention.

### Impact on Patient Experience

Clinical outcomes in CKD trials (as with all trials) are defined as “how a person feels, functions, or survives.”^77^ Therefore, in addition to (overall or kidney) survival, how a patient functions or feels from day to day is an important consideration. However, data on the disease-specific symptom burden for people living with IgAN and data from clinical trials on patient-reported outcomes in IgAN are currently limited (Table 1),^78^, ^79^, ^80^ meaning that such measures cannot currently be used to inform disease modification in IgAN. It is hoped that data relating to a patient’s direct experience will be forthcoming in future studies, irrespective of whether the investigational agents are addressing the IgAN-specific, immune-mediated nephron loss directly or the generic maladaptive responses to IgAN-induced nephron loss. This was clearly advocated for by patients when they were asked what outcomes should be evaluated when assessing new interventions, as part of the Standardized Outcomes in Nephrology–Glomerular Diseases study.^81^

Although data on patient-reported outcomes and quality of life in IgAN remain limited, available studies demonstrate that patients experience a substantial burden of symptoms—including fatigue, edema, and impaired life participation—that have a major impact on daily functioning and wellbeing.^82^ To date, most trials have relied on generic patient-reported outcome instruments, and there is currently no validated IgAN-specific tool, thereby complicating the assessment of meaningful disease modification from the patient’s perspective.^83^ Consensus efforts such as the Standardized Outcomes in Nephrology–Glomerular Diseases initiative have emphasized the need to include core patient-centered outcomes such as life participation and symptom burden, in all future IgAN trials to ensure that research addresses what matters most to patients and supports holistic, patient-centered care.^81^

### Rate of Change in eGFR

By definition, a decline in eGFR is on the pathway to kidney failure (eGFR < 15 ml/min per 1.73 m^2^),^13^^,^^77^ and there is a strong consistent relationship between eGFR decline and kidney failure and death.^13^^,^^84^ An eGFR decline ≥ 30% is used as a surrogate end point for progression to kidney failure in patients with CKD^13^^,^^77^; however, this is of limited use in either clinical practice or IgAN clinical trials, where long follow-up durations would be needed to detect this degree of eGFR change. The eGFR slope may be more practical to measure in this context, because it has been established as a surrogate end point in recent years.^85^, ^86^, ^87^ A meta-analysis of clinical trials in IgAN reported a positive relationship between treatment effects on eGFR slope and proteinuria, both when considering total eGFR slope (from randomization to 1, 2, and 3 years) and chronic eGFR slope (from 3 months after randomization to 1, 2, and 3 years).^10^ In a separate meta-analysis, 1-year eGFR slope was shown to be an independent predictor of treatment effect on long-term clinical outcomes in IgAN (interestingly, the same study did not find proteinuria reduction at ≤ 1 year to be an independent predictor once the impact on 1-year eGFR slope had been considered).^88^ Interventions targeted at either the IgAN-specific or the generic CKD pathways driving nephron loss would be expected to have a direct impact on eGFR slope. Therefore, a sustained effect on eGFR slope, assessed over a period of ≥ 2 years, would be an appropriate indication of a treatment strategy’s disease-modifying effect in IgAN. Although current clinical trials are examining changes in eGFR slope over 2 years, the time frame over which eGFR slope is assessed may vary in clinical practice and is likely to be much longer.

### Changes in Albuminuria and Proteinuria

Reductions in albuminuria and proteinuria are defined treatment goals in patients with CKD.^77^^,^^89^^,^^90^ Both proteinuria (which is universally measured in IgAN studies) and albuminuria (which, in contrast, is not often measured in IgAN studies) may be driven by both IgAN-specific immune-mediated drivers of nephron loss and the maladaptive generic responses to nephron loss in IgAN. There is also considerable evidence that in addition to being biomarkers of glomerular injury, albuminuria and proteinuria contribute to downstream kidney inflammation; this is believed to occur through multiple mechanisms, including endothelial dysfunction as well as chemokine and complement activation in the tubulointerstitial compartment.^20^^,^^91^^,^^92^

Albuminuria is considered a biomarker of kidney damage in patients with CKD, with an increase in urinary albumin excretion identified as a strong predictor of progression to kidney failure.^89^^,^^93^ In recent years, meta-analyses have identified early change in albuminuria as a surrogate end point for the progression of kidney disease in clinical trials.^94^^,^^95^ However, observational studies^96^^,^^97^ and clinical trials (Table 1) providing support for albuminuria as a surrogate end point in IgAN are currently limited; this is possibly because of the early establishment of urinary protein excretion as a biomarker of IgAN progression.^98^ More research is needed to determine whether albuminuria can be used as a surrogate biomarker of disease modification in patients with IgAN.

Proteinuria is a well-established biomarker of kidney damage; both cohort study and trial-level analyses in patients with IgAN have reported a strong association between changes in proteinuria and kidney outcomes (e.g., kidney failure), independent of the intervention employed.^70^^,^^98^^,^^99^ As a result, proteinuria reduction is considered a “reasonably likely” surrogate end point for progression to kidney failure in IgAN.^10^^,^^99^ The 2021 Kidney Disease: Improving Global Outcomes guidelines recommended a treatment target for proteinuria < 1 g/d in IgAN,^14^ although individuals who meet this target may still have a high lifetime risk of kidney failure.^5^

It is important to acknowledge that albuminuria and proteinuria can result from both the IgAN-specific immune-mediated and generic CKD-related drivers of nephron loss (and be positively impacted by interventions in both of these areas), as well as by irreversible glomerular scarring and the loss of the protein resorptive capacity of the nephron because of tubular loss (which will not be impacted by therapeutic interventions).^18^^,^^100^^,^^101^ Furthermore, reductions in albuminuria and proteinuria do not provide a great deal of insight into the mechanism(s) of action of a drug.

### Impact on Nonvisible Hematuria

Although not unique to IgAN, nonvisible (or microscopic) hematuria is the most common clinical feature of the disease.^102^ It may itself be a contributory factor to disease progression through hemoglobin-induced oxidative damage.^103^ Hematuria results from damage to the glomerular basement membrane, which can be a consequence of active inflammation or from ongoing glomerular scarring in the absence of inflammation.^102^^,^^104^ Therefore, it may be driven by both the IgAN-specific, immune-mediated causes of nephron loss and the generic maladaptive responses to nephron loss. In turn, it may also be influenced by treatments acting on IgAN-specific and generic pathways.

The value of measuring hematuria to monitor disease progression and treatment effect is debated. For example, there has been a lack of standardization between centers regarding its measurement, and there is considerable variability in the day-to-day levels of hematuria that complicates interpretation.^102^ As a result, hematuria often only features as an exploratory end point in large-scale clinical trials (Table 1).

### Impact on Components of the IgAN Pathogenic Cascade

A number of serum, urinary, and kidney biopsy biomarkers may potentially be used to measure the disease-modifying impact of an agent at ≥ 1 “hits” of the cascade. For example, reductions in the levels of circulating Gd-IgA1 at “Hit 1,” anti–Gd-IgA1 IgA and IgG antibodies at “Hit 2,” immune complexes at “Hit 3,” and urinary biomarkers of glomerular inflammation and complement activation at “Hit 4,” alongside repeat kidney biopsy evaluation, have each been reported in studies to indicate disease modification.^27^^,^^36^^,^^52^^,^^105^, ^106^, ^107^, ^108^

These biomarkers provide a mechanistic framework for assessing whether a therapeutic intervention is influencing the underlying immunopathogenesis of IgAN rather than merely mitigating downstream consequences such as proteinuria or reduced eGFR.^109^ For example, the ability of an agent to lower Gd-IgA1 levels suggests an upstream effect on IgA1 glycosylation pathways, potentially reflecting alteration in B-cell function or mucosal immune responses.^109^ Similarly, decreases in anti–Gd-IgA1 autoantibodies may point to modulation of the adaptive immune response, potentially through B-cell depletion or inhibition of autoantibody-producing plasma cells.^109^ A reduction in circulating immune complexes may represent a composite effect on both antibody generation and complex formation, suggesting a deeper attenuation of the systemic autoimmune process.

Emerging data suggest a correlation between proximal “hit” biomarkers and clinical end points such as proteinuria.^40^ For example, in analyses of interventional cohorts, reductions in Gd-IgA1 and circulating immune complexes were noted.^27^^,^^32^^,^^36^^,^^40^ However, these associations remain observational and causality has not been established.

Downstream markers such as urinary monocyte chemoattractant protein-1, CD163, or components of the alternative and lectin complement pathways (e.g., Ba, mannose-binding lectin-associated serine protease 2) may serve as indicators of intrarenal inflammation and complement-mediated injury.^110^, ^111^, ^112^, ^113^ These provide insights into the degree of immune activation and glomerular injury occurring within the kidney, even in the absence of overt changes in serum creatinine or proteinuria.

Few studies have evaluated the impact of immunosuppressive therapies on kidney histology in IgAN using repeated biopsies. Two trials assessing immunosuppressive therapy (including mycophenolate mofetil) demonstrated reductions in active lesions such as endocapillary hypercellularity and crescents,^107^^,^^108^ though one trial reported worsening tubulointerstitial damage.^108^ A larger retrospective study involving 168 patients found that tubulointerstitial lesions worsened over time regardless of treatment, and changes in crescents and tubular atrophy were linked to progression to CKD.^114^ Overall, repeated biopsy studies show a reduction in active inflammatory lesions but a tendency toward progressive chronic tubulointerstitial damage.^107^^,^^108^^,^^114^

It is important to note that the reported “hit”-related biomarker data are currently limited (Table 1) and that there are no prospective data at present linking changes in these biomarkers to clinical outcomes in IgAN. However, these are biologically plausible biomarkers of IgAN-specific, immune-mediated disease activity, and each warrants further investigation to confirm its relevance to clinical outcomes. Importantly, if these biomarkers are to enter clinical practice and be used as part of routine clinical care to document disease modification, standardized and approved assays must be developed.

## Conclusion

It is the authors’ view that to be considered “disease modifying” in IgAN, any intervention needs to address the “progressive component or the natural course of the disease,” which is the gradual loss of nephrons over time, with the aim of avoiding progression to kidney failure.

For those interventions purporting to address the IgAN-specific, immune-mediated drivers for nephron loss, it is the authors’ view that to be considered disease-modifying, a statistically significant effect on rate of loss of eGFR (or kidney failure end points) in phase 3 must be accompanied by significant changes in biologically plausible biomarkers in the same clinical trial, reflecting a direct impact on the pathogenic “hits” of IgAN. Importantly, because of the significant heterogeneity of IgAN in individuals of different racial or ethnic backgrounds and ages,^4^^,^^5^ if possible, these clinical trials should be representative of the global IgAN population and ideally examine the ability of any agent to modify IgAN-specific, immune-mediated drivers for nephron loss in different racial/ethnic and age groups.

For those interventions targeting the generic responses to IgAN-induced nephron loss seen in other forms of CKD**,** there should be data available from well-conducted phase 3 trials, ideally in patients with IgAN, which show a statistically significant benefit favoring the experimental treatment on the rate of loss of eGFR (or kidney failure end points). However, eGFR or other kidney failure end point data from the broader CKD population may be appropriate to conclude a disease-modifying action in IgAN if the recruited population contains a sizeable number of patients with IgAN. One example of this would be the analysis of the effect of sodium-glucose cotransporter 2 inhibition on kidney function in a subpopulation of patients with IgAN.^60^

It is important to note that as proteinuria (and nonvisible hematuria) is driven by both IgAN-specific immune-mediated glomerular injury and the consequent generic maladaptive responses to nephron loss, its reduction is likely to accompany both types of intervention. Emerging data, however, suggest that the relationship between the extent of early proteinuria reduction and future kidney function loss (nephron preservation) may depend on the site of action of the drug. Potentially, treatments targeting the IgAN-specific, immune-mediated disease activity may deliver greater nephron protection for the same degree of proteinuria reduction than treatments targeting the generic maladaptive responses to IgAN-induced nephron loss.^25^^,^^32^^,^^36^ This, however, remains to be proven. As more data become available from randomized controlled trials, it will be possible to compare the magnitude of proteinuria reduction by a given class of drug with the degree to which that reduction translates into eGFR protection over the 2 years of the clinical trial. These data will be augmented with longitudinal real-world data. In addition, biomarker studies and, where available, repeat kidney biopsy studies offer the opportunity to see how different classes of drugs impact on the fundamental pathophysiology of IgAN, and whether drug classes targeting specific aspects of disease pathogenesis deliver more or less long-term protection against eGFR decline.

IgAN is a chronic disease that invariably leads to progressive loss of kidney function, resulting in reduced quality of life; treatments that demonstrate a clear disease-modifying effect in clinical trials and in the real world are of key importance. The considerations provided here are those of the authors and are in no way intended to be definitive. However, the intention is to provide the necessary overview to begin meaningful discussions on what “disease-modifying” treatment in IgAN looks like. It is our belief that a combined strategy that intervenes both in the disease-specific, immune-mediated mechanisms of nephron loss and the generic CKD-related responses to nephron loss in IgAN is needed to improve the prospects of patients with IgAN and prevent kidney failure within their lifetime. A better understanding of how each intervention modifies nephron loss in IgAN will enable clinicians to identify which therapies should be combined to achieve the greatest degree of nephron protection. To facilitate this, agreed surrogate clinical measures and validated IgAN-specific biomarkers are needed.

## Disclosure

JB is a consultant for Alebund, Alnylam Pharmaceuticals, Alpine, Argenx, Astellas, BioCryst, Calliditas Therapeutics, Chinook, CSL Vifor, Dimerix, Hi-Bio, Kira, Novartis, Omeros, Otsuka, Q32 Bio, Roche, Sanofi, Takeda, Travere Therapeutics, Vera Therapeutics, and Visterra; and has received research funding from Argenx, Calliditas Therapeutics, Chinook, Galapagos, GlaxoSmithKline, Novartis, Omeros, Travere Therapeutics, and Visterra. LHM reports receiving consulting fees from Calliditas Therapeutics, Dimerix, Novartis-Chinook Therapeutics, Travere Therapeutics, and Vera Therapeutics; and receiving grant support to her institution from Boehringer Ingelheim, Calliditas Therapeutics, Hi-Bio, NephCure Kidney International, Reliant Glycosciences, Takeda Therapeutics, and Travere Therapeutics.

JR reports receiving research grants from Travere Therapeutics; and consulting fees from Alexion Pharmaceuticals, Apellis Pharmaceuticals, Calliditas Therapeutics, and Otsuka Pharmaceuticals. He has been a Data Safety Monitoring Board member for Novartis and a steering committee member for Alexion Pharmaceuticals and Travere Therapeutics. He was Editor-in-Chief of *Kidney International Reports* at the time of manuscript submission. DVR has grants (pending/received) and research support from Reata Pharmaceuticals, Travere Therapeutics (Retrophin), Calliditas Therapeutics (Pharmalink), Otsuka Pharmaceuticals (Visterra), Vertex Pharmaceuticals, Chinook Pharmaceuticals, Vera Therapeutics, and LaRoche. She has received consulting fees from Novartis, George Clinical, Eledon Pharmaceuticals, Otsuka Pharmaceuticals (Visterra), Calliditas Therapeutics (Pharmalink), Chinook Pharmaceuticals, LaRoche, Vera Therapeutics, BioCryst, Chugai, Biogen, and Timberlyne Therapeutics; and honoraria from Calliditas Therapeutics (Pharmalink), Chinook Pharmaceuticals, Otsuka Pharmaceuticals, Vera Therapeutics, BioCryst, Argenx, Alpine Immune Science, and GlaxoSmithKline. She also has part-ownership in Reliant Glycosciences LLC. JAT reports grant support from AstraZeneca, Bayer, Calliditas Therapeutics, Chinook Therapeutics, Dimerix, George Clinical, Novartis, Omeros, Otsuka Pharmaceuticals, and Vera Therapeutics; consulting fees from AstraZeneca, Biogen, Calliditas Therapeutics, Chinook Therapeutics, Dimerix, George Clinical, Novartis, Omeros, Takeda Pharmaceuticals, Travere Therapeutics, and Vera Therapeutics; honoraria from AstraZeneca, Calliditas Therapeutics, Chinook Therapeutics, George Clinical, Novartis, and Travere Therapeutics. RAL reports institutional grants from Beigene, Calliditas Therapeutics, ChemoCentryx, Omeros, Otsuka Pharmaceuticals, Pfizer, Roche, Travere Therapeutics, Vera Therapeutics, and Visterra; he has received consulting fees from Alexion Pharmaceuticals, Beigene, Calliditas Therapeutics, Omeros, Otsuka Pharmaceuticals, Travere Therapeutics, Vera Therapeutics, and Vertex; and has served on advisory boards for Cara Therapeutics.
